# Mass‐Spectrometric Imaging of Electrode Surfaces—a View on Electrochemical Side Reactions

**DOI:** 10.1002/anie.202010134

**Published:** 2020-09-02

**Authors:** Jens Fangmeyer, Arne Behrens, Barbara Gleede, Siegfried R. Waldvogel, Uwe Karst

**Affiliations:** ^1^ Institute of Inorganic and Analytical Chemistry University of Münster Corrensstrasse 30 48149 Münster Germany; ^2^ Department of Chemistry Johannes Gutenberg University Mainz Duesbergweg 10–14 55128 Mainz Germany

**Keywords:** electrochemical side reactions, electrochemistry, electrode fouling, MALDI-MS imaging, polymerization

## Abstract

Electrochemical side reactions, often referred to as “electrode fouling”, are known to be a major challenge in electro‐organic synthesis and the functionality of modern batteries. Often, polymerization of one or more components is observed. When reaching their limit of solubility, those polymers tend to adsorb on the surface of the electrode, resulting in a passivation of the respective electrode area, which may impact electrochemical performance. Here, matrix‐assisted laser‐desorption/ionization mass spectrometry (MALDI‐MS) is presented as valuable imaging technique to visualize polymer deposition on electrode surfaces. Oligomer size distribution and its dependency on the contact time were imaged on a boron‐doped diamond (BDD) anode of an electrochemical flow‐through cell. The approach allows to detect weak spots, where electrode fouling may take place and provides insight into the identity of side‐product pathways.

## Graphical abstract

In‐depth understanding of electrochemical side reactions is essential for the optimization of new electrode materials or electrochemical cell geometry. The presented MALDI‐MS imaging approach offers a fast and elegant way to detect electrochemical byproducts on the electrode surface and provides additional information about molecular mass and spatial distribution.

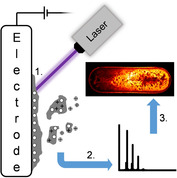

In recent years, electrochemistry became an important and permanently growing topic not only in the access to new molecular structures via electro‐organic synthesis,^[1]^ but also in battery research^[2]^ and in the treatment of wastewater.^[3]^ The first has evolved as a powerful tool in modern organic synthesis and is applied for several reaction types, which has been currently reviewed.^[4]^ Also, it can be considered as a part of green chemistry and therefore reducing the ecological footprint of chemical processes when renewable electricity is utilized.^[5]^ Electrochemical conversions are typically initiated by single‐electron‐transfer reactions (SET), which are then followed by functionalizations.^[6]^ Besides the desired reaction, the initial radical derived from SET reactions may undergo side reaction pathways such as dehydrodimerization or higher polymerization.^[7]^ In that case, formed oligomers may exceed their solubility in the solvent used, leading to precipitation and adsorption on the respective electrode surface. As a result, deposited material compromises the efficacy of the conversion because of passivation of the electrochemically active surface, which is predominantly known as electrode fouling. This is not only a common concern in electro‐organic synthesis, but also in electroanalytical chemistry (Scheme 1).^[8]^

**Scheme 1:**
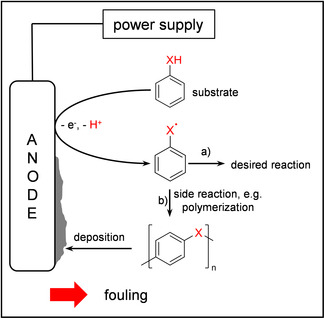
Overview of the electrochemical conversion in conventional electro‐organic synthesis routes. Route a) represents the desired reaction pathway while b) describes possible side reactions such as polymerization, which might occur during the synthesis and result in passivation of the surface.

In the past, electrode fouling has been observed at different electrode materials such as platinum, glassy carbon or even boron‐doped diamond (BDD).[^7^, ^8^, ^9^] In case of BDD, fouling processes can significantly be avoided if higher potentials are applied and water is present,[^4^, ^10^] since hydroxyl radicals may be formed, which may suppress the formation of polymeric thin layers by self‐cleaning. Nevertheless, very positive working electrode potentials can be applied,^[6]^ but may limit the electrochemical selectivity towards certain reaction types.^[11]^

Hanssen et al. recently reviewed the wide field of lately developed strategies in fouling minimization.^[12]^ In addition, coating the electrodes by such processes might enhance the selectivity.^[13]^ Therefore, analytical techniques, which provide deeper insight into fouling processes are essential for the optimization of electrochemical reactions with respect to electrode material and shape, but also the overall cell geometry.

In the past, techniques like atomic force microscopy (AFM), scanning electrode microscopy (SEM), Raman and Fourier‐transform infrared (FTIR) spectroscopy or voltammetry techniques were utilized for the investigation of electrode fouling.[^7^, ^8^, ^9^, ^14^] All of them have provided valuable information in the chemical functionalization of the electrode surface, but none of them provides detailed insight in the molecular composition of the surface layer. While on‐line mass spectrometry has been used frequently for the molecular investigation of electrochemical generated products in the cell effluent, it has not yet been applied on the electrode surface.^[15]^ In this work, we present matrix‐assisted laser desorption/ionization mass‐spectrometry imaging (MALDI‐MSI) as a promising technique to visualize and investigate polymer‐based electrode fouling on working electrodes. In the 1970s, Hillenkamp et al. used laser desorption ionization (LDI) for the first spatial analysis of small molecules via mass spectrometry.^[16]^

The technique was further developed by the use of matrix application and hence enabled access to larger (bio)molecules such as polymers and proteins.^[17]^ In recent years, MALDI‐MSI has established as powerful tool for chemical imaging of tissues of human, animal or plant origin.^[18]^ To the best of our knowledge, within this work MALDI‐MS is applied to the imaging analysis of electrode surfaces for the first time.

We investigated the oxidative electrochemically induced polymer formation of aniline, 4‐ethylphenol and *o*‐phenylenediamine (Scheme 2), either bearing common functional groups or belonging to the group of classical building block chemicals. All compounds were oxidized within an electrochemical thin‐layer flow‐through (EC) cell (μ‐PrepCell 2.0, Antec Scientific, Zoeterwoude, The Netherlands) under potentiostatic control using an in‐house developed potentiostat. Therefore, a three‐electrode setup consisting of a changeable BDD working electrode (WE), a graphite‐doped conductive polyetheretherketone (PEEK) counter electrode (CE) and a Pd/H_2_ reference electrode (RE) was used. A schematic overview of the setup is shown in the right part of Scheme 2. For polymer formation, the respective compound solutions were passed through the electrochemical cell with a constant flow rate of 10 μL min^−1^. All investigated electrodes were analyzed with the timsTOF fleX (Bruker Daltonik GmbH, Bremen, Germany). Detailed mass spectrometric parameters as well as detailed sample preparation procedures are presented in the SI.

**Scheme 2:**
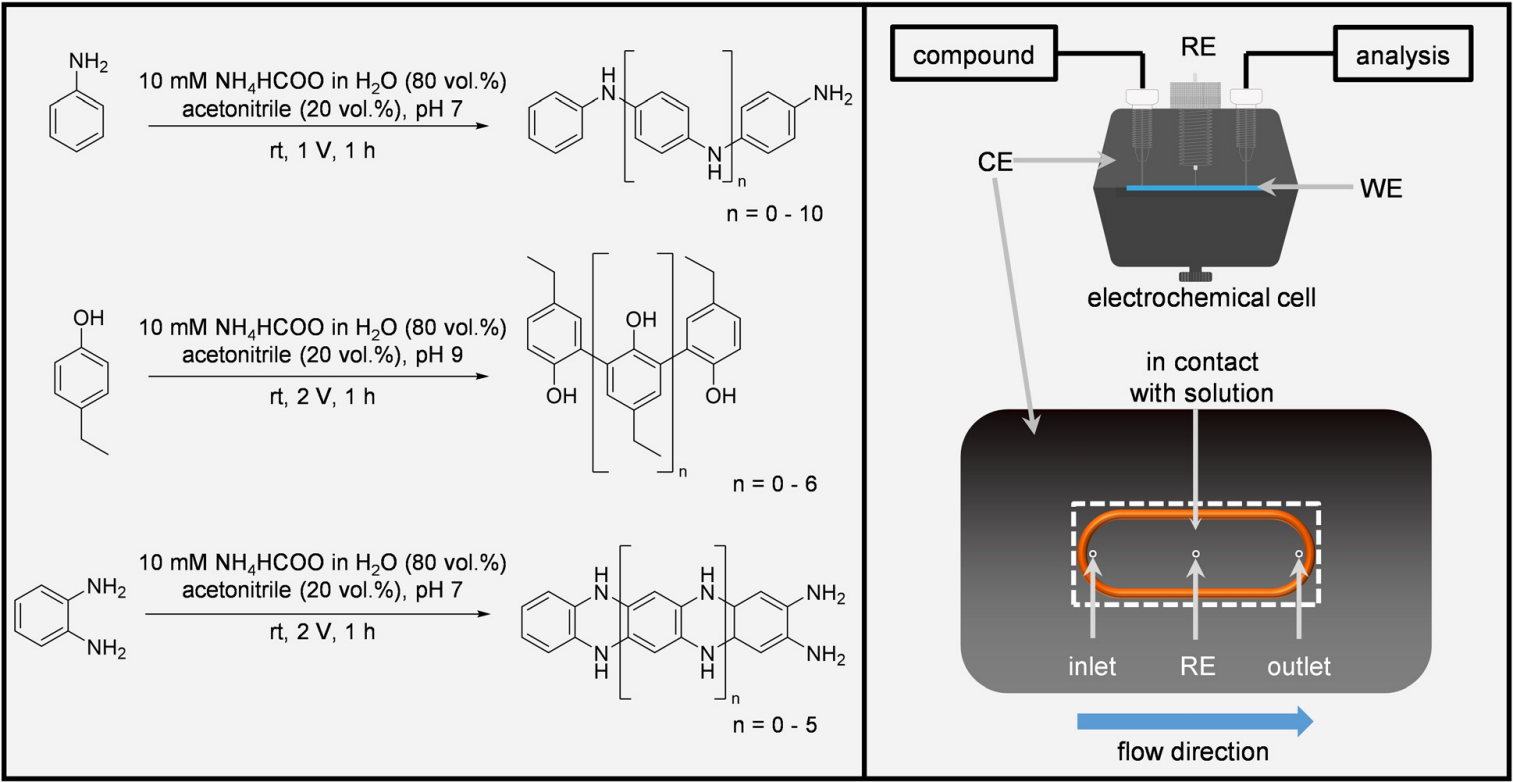
Left: Polymerized organic compounds and respective electrochemical conversion parameters. Each compound was dissolved in the respective solvent mixture resulting in a total concentration of 10 mm. The presented structures are examples from a large variety of different possible isomers of the observed products. Right: Overview of the electrochemical three‐electrode setup. The lower part of the Scheme shows the CE of the EC cell. The orange O‐ring encloses the region, where the CE and the WE are in contact with the respective compound solution. The three circles represent three different channels belonging to the inlet, the outlet and the connection to the RE. The dashed rectangle represents the area covered by the BDD WE (12×30 mm).

First, the oxidation potential delivering the highest conversion was identified for each of the three investigated compounds (Figures SI‐3a–c). Subsequently, this potential was applied in a flow‐through experiment for one hour with solutions of 10 mm concentration, and the solution containing the products was collected. An aliquot of this solution was analyzed via direct infusion to obtain an overview about the detectable oligomers in solution. The respective mass spectrum (Figure SI‐4a) shows, in case of aniline, a high signal intensity for the dimeric species and only a low one for the trimers. For 4‐ethylphenol (Figure SI‐4b) and *o*‐phenylenediamine (Figure SI‐4c), oligomers with up to four monomeric units were detected. Higher oligomers cannot be observed leading to the conclusion that either those compounds are not formed, or they are not present in the oxidized solution. As a result, we concluded that higher oligomers likely exceed their individual solubility and may therefore adsorb on the electrode surface. In the next step, dried droplet experiments were conducted to check overall MALDI applicability and therefore, different matrices were evaluated. Besides the compound specific electrochemical parameters, all experiments were performed under equal conditions, by using the left side of the cell as input channel which results in a flow from the left to the right side. Afterwards, the remaining reaction solution on the BDD electrodes was washed off with bidistilled water after careful disassembly of the EC cell. In case of aniline, 2,5‐dihydroxybenzoic acid (DHB) was applied as matrix with a commercially available TM‐Sprayer (HTX Technologies LLC, Chapel Hill, NC, USA). The other two compounds were analyzed without any applied matrices, since 4‐ethylphenol and *o*‐phenylenediamine show excellent UV/vis absorbance during dried droplet experiments. Therefore, both were analyzed by direct laser desorption/ionization (LDI) mass‐spectrometry.

After (MA)LDI analysis of the respective BDD electrodes, the averaged spectra of the active electrode area were plotted for each of the three compounds and electrodes (Figure 1). Due to the selected mass spectrometric parameters, the monomeric species are suppressed and therefore not detectable. Hence, oligomers with a size of up to dodecamers can be detected in case of aniline with the applied method. With increasing oligomer size, the corresponding number of signals increases for each oligomer fraction. Additionally, hydroxylated species can be detected for 4‐ethylphenol and its respective oligomers. For all compounds, the generation of different ions including protonated analytes and/or molecular cations/anions is observed. In addition to that, long oligomeric chains or even cyclic structures can be formed. According to literature, a vast amount of combinations and even condensation reactions are possible for all the compounds, which result in broader signal groups for higher oligomer sizes.[^14^, ^19^] Figure 1 also shows photographic images of the three BDD electrodes made after cell dissembling and prior to matrix application. In case of aniline and *o*‐phenylenediamine, only minor color changes due to polymer deposition can be observed. These are located closely to the border of the active electrode area or close to the outlet of the cell. In contrast to this, 4‐ethylphenol shows intense adsorption on the respective electrode surface indicated by strong darkening in flow direction. The SCiLS lab software (Bruker Daltonik GmbH, Bremen, Germany) enabled the visualization of the spatial oligomer distribution. For each oligomer size one respective *m*/*z* was plotted. In case of 4‐ethylphenol, we also present the image of the hydroxylated dimer.

**Figure 1:**
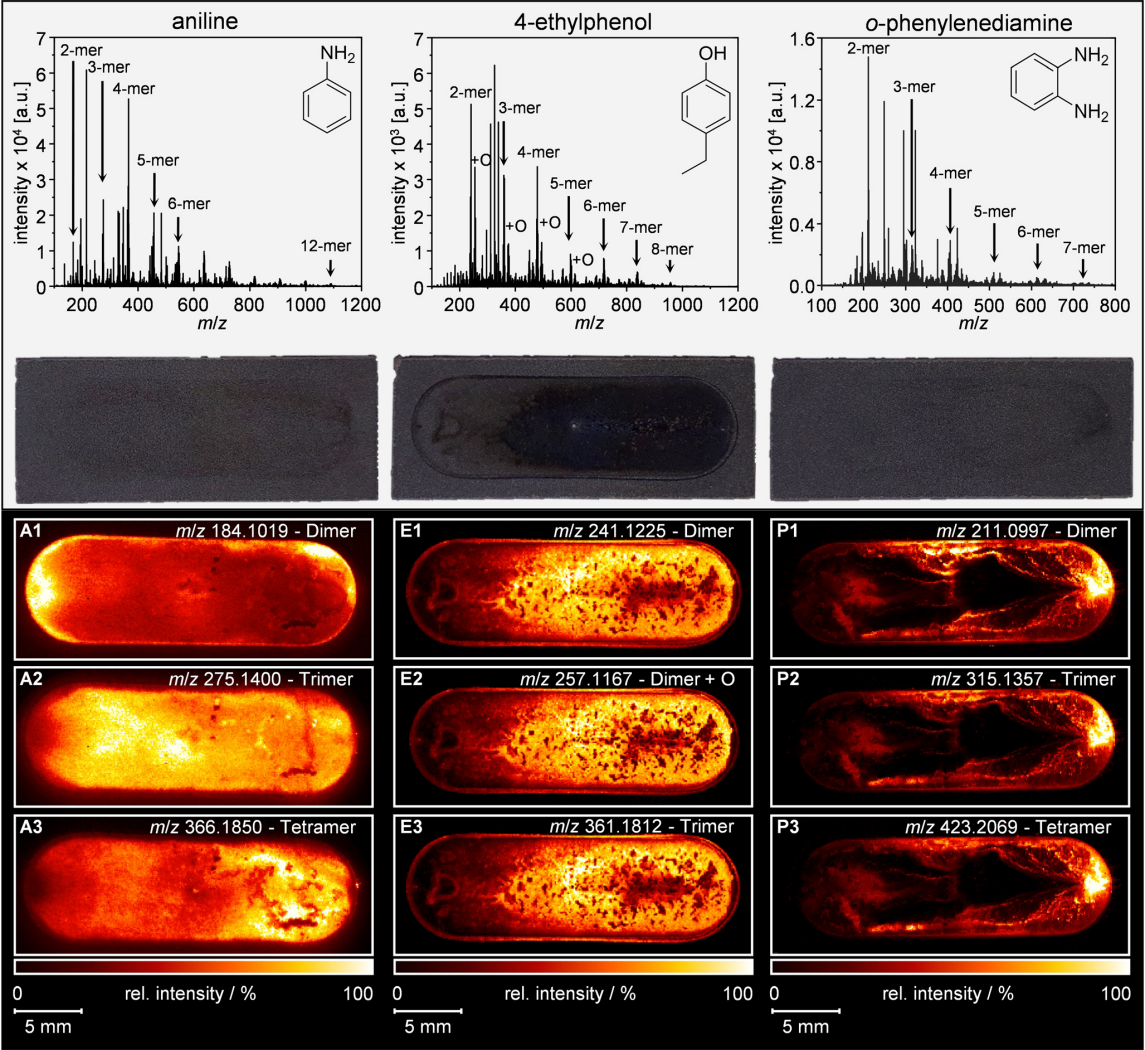
General: The results for the three compounds are presented column‐wise. Top row: Mean mass spectra for each BDD electrode of the three investigated compounds. Second row: Photographic images of the three electrodes after the electrochemical treatment. Below: (MA)LDI‐MS images of the spatial oligomer distribution of oxidative polymerized aniline (A1–3), 4‐ethylphenol including one hydroxylated compound (E1–3) and *o*‐phenylenediamine (P1–3). The flow direction for the electrochemical conversion is from the left to the right.

The images for the polymerization of aniline are shown on the left side in Figure 1. The three illustrated oligomers types show a different spatial distribution on the electrode surface. Since aniline already tends to form dimers without external voltage application due to autoxidation, the respective signal hotspot close to the inlet region can be explained. Additionally, the aniline dimer shows significantly higher intensities in the region of the outlet channel of the cell due to longer contact time with the electrode (Figure 1 A1). With increasing oligomer size, the highest signal intensity of the respective oligomer moves more to the right of the electrode, which correlates well with the contact time and therefore the oxidation time. As a result, tetrameric aniline can be found predominantly close to the outlet region of the electrode surface (Figure 1 A2,3).

For 4‐ethylphenol, the conditions of our experiments enable oligomer formation up to octamers. Besides the different oligomers, we also observed hydroxylations after oxidative treatment. Figure 1 E1–3 illustrates the spatial distribution of dimeric, hydroxylated dimeric and trimeric structures, which show a perfect correlation of their intensity with the flow direction. An area of lower oligomer abundancy is in the center of the last third of the electrode, while higher signal intensities are observed close to the edge of the reactive surface. Comparing the three *m*/*z* images with the photograph, a match of the observed adsorption pattern can be seen.

For *o*‐phenylenediamine, oligomers of up to seven units were detected (Figure 1). The respective images of dimeric, trimeric, and tetrameric structures generally show an almost identical distribution pattern (Figure 1 P1–3). Hotspot regions of all three oligomers can be identified predominantly close to the outlet region of the EC cell. Additionally, higher signal intensities are located at the edges of the active electrode surface, whereas low intensities are recorded in the center of the electrode. Furthermore, the dimer shows a significantly increased signal intensity at the half distance from inlet to outlet at the border of the electrode. The same phenomenon is found for the tetramer at the lower part of the respective image (Figure 1 P3).

For 4‐ethylphenol and *o*‐phenylenediamine, we concluded that first, precipitation results in conductive polymeric thin layers, which may then act as condensation nuclei where new oligomers can sprout. In that case, this process would be kinetically preferred towards the direct formation of higher oligomers on the electrode surface itself.

While parts of the findings can be explained by electrode geometry and consideration of the reaction conditions, the large data sets obtained have the potential to provide much more insight into electrode (side) reactions and their optimization.

The reported results were additionally confirmed by a real‐life example from electro‐organic synthesis, which is well‐known from literature.[^5^, ^20^] For this purpose, the cross‐coupling reaction of phenols was used (Figure 2). For a successful dehydrogenative cross‐coupling reaction, the phenol A with the lower oxidation potential is initially oxidized at the BDD anode. The resulting phenoxyl radical is then attacked nucleophilically by phenol B. After a second oxidation step at the anode, the desired biphenol is obtained. It is crucial to have control over the oxidation steps at the electrode surface as well as the electrolyte‐controlled follow‐up reaction.^[21]^ The high selectivity of the synthesis is achieved by using 1,1,1,3,3,3‐hexafluoropropan‐2‐ol (HFIP), especially in combination with additive such as water or methanol to suppress successfully side reactions, for example, homo‐coupling and minimize over‐oxidation.^[22]^ Due to the electrode dimensions, it was mounted in an in‐house built adapter for subsequent analysis. Based on the results of 4‐ethylphenol, the phenol‐phenol cross‐coupling was analyzed under the same mass spectrometric conditions in the negative LDI‐MS mode without the application of an additional matrix.

**Figure 2:**
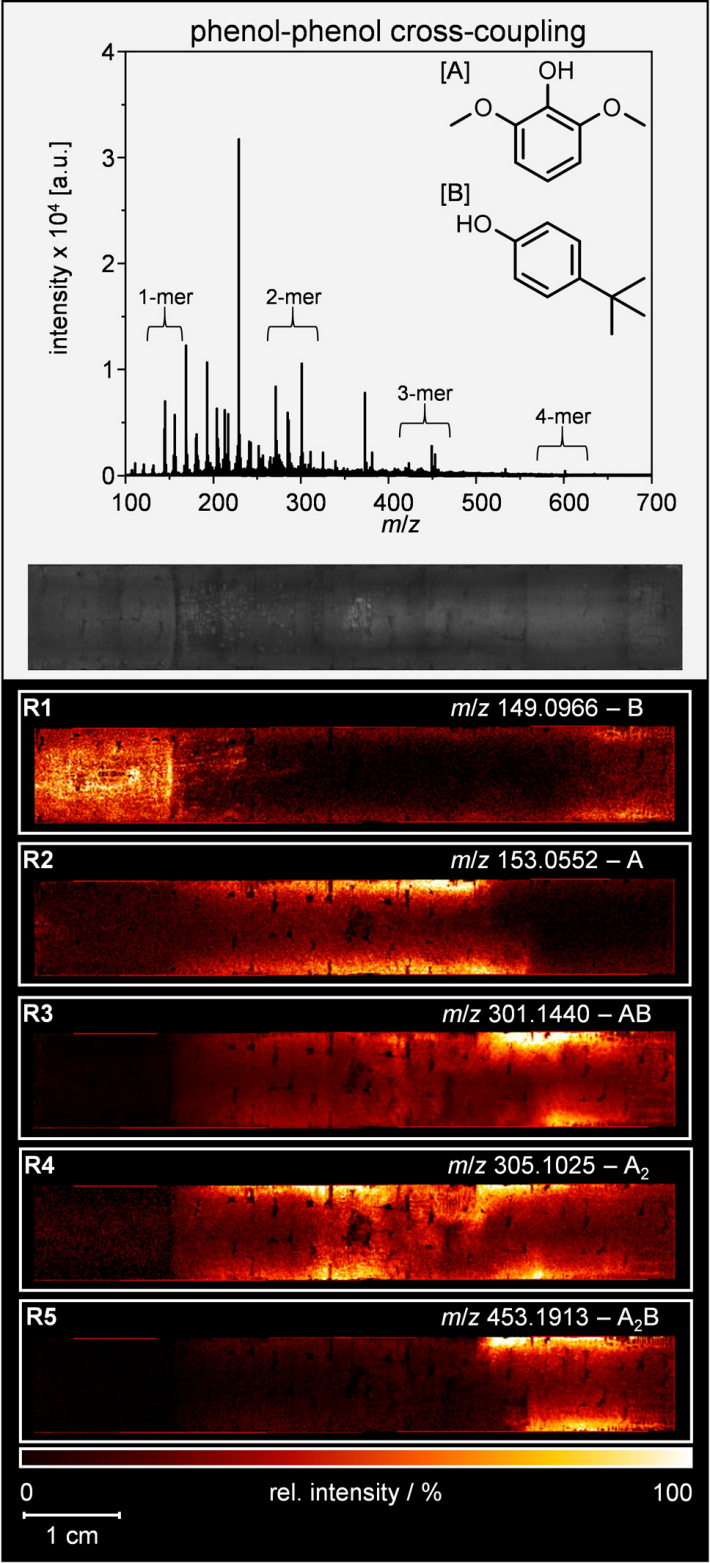
Mean mass spectrum (negative ion mode) for the BDD electrode of the phenol‐phenol cross‐coupling reaction. Optical image of the electrode after electrochemical treatment. Below: Selected LDI‐MS images of the spatial monomer and oligomer distribution of oxidatively treated 2,6‐dimethoxyphenol (A) and 4‐(1,1‐dimethylethyl)‐phenol (B; R1–5). The flow direction for the electrochemical conversion is from the left to the right.

Again, the average spectrum of the active electrode surface was plotted (Figure 2). In this case, monomeric species as well as oligomeric species up to dehydropentamers with different connectivity of A and B were observed mainly as deprotonated ions. These include their respective oxidation products like hydroxylated, dehydrogenated or demethylated species (Figure SI‐6). The optical image in Figure 2 shows a darkening of the anode surfaces towards the edges and a vertical line after the first fifth of the electrode. Additionally, indentations in the electrode surface can be observed due to the nature of the graphitic support material.

Distributions for selected monomeric, dehydrodimeric, and dehydrotrimeric species are visualized in Figure 2. Starting material B is mainly localized in the first part of the electrode. This region is confined by the dark vertical line in the optical image. The signal intensities for B vertically increase towards the mid of the electrode (Figure 2 R1). In contrast, educt A was only found in the electrode's midsection with a vertical increase towards the edges (Figure 2 R2). Towards the end of the electrode, the signal for A shows an abrupt decrease, while the signal for the dehydrodimeric product AB reaches its maximum. The formation of AB can already be observed with the inlet of A (Figure 2 R3). Nevertheless, an intense deposition of AB is only observed in a rather small area and fading again towards the end of the electrode. The formation of higher oligomers is exemplary depicted by the formation of A_2_B (Figure 2 R5). As its deposition abruptly begins with the hotspot region of AB and fades out towards the end, it indicates a reaction of the AB species with an additional A monomer. Depositions of further functionalized A_2_B‐type oligomers, for example, dehydrogenated or hydroxylated species, can be found in the midsection of the electrode as well (Figure SI‐6). Hence, an alternate reaction of B with functionalized A_2_‐type dehydrodimers seems to dominate here. The identified mixed dehydrotrimers of both components are in‐line with polycyclic compounds observed previously. Such products might be over‐oxidized by hydroxyl radicals causing an additional installation of oxygen.^[23]^

Dehydrodimeric A_2_ is formed immediately with the inlet of starting material A and is intensified towards the electrodes center (Figure 2 R4). This hotspot region persists until the formation of AB surpasses. Here, a curve in the flow profile leading to an increased dead volume of the cell becomes apparent shortly after the center of the electrode. All images show identical blind spots correlating with the indentations observed in the optical image. In these concavities the electrodes surface is not accessible for the laser beam and, therefore, for mass spectrometric analysis.

In summary, the phenol‐phenol cross‐coupling reaction of the phenolic educts A and B to their dehydrodimeric product AB was visualized on a synthetic BDD electrode. Additionally, the formation and reaction pathways of side reactions based on over‐oxidation can be depicted as well as possible weak spots in the overall cell design.

To conclude, (MA)LDI‐MS was presented as valuable analysis technique for the visualization of electrochemical polymerization side reactions. Therefore, the electrochemical polymerization of aniline, 4‐ethylphenol and *o*‐phenylenediamine was investigated, all of which show significant polymer formation during electrochemical oxidation on BDD electrodes. The presented method therefore is a promising tool for the optimization of electro‐organic reactions, since occurring side reactions can be determined and localized rapidly and easily. Thereby, efficacy and reproducibility of the electrochemical processes can be increased. Especially the fields of electro‐organic synthesis and battery research might benefit from this approach since their methods and techniques can be optimized thoroughly with the support of the presented (MA)LDI‐MS approach. In this case, the surface of BDD electrodes was investigated, but the method allows to address all planar electrodes independent of the electrode material. Additionally, this method provides accurate molecular mass information plus the option for data dependent fragmentation, which allows to identify the individual compounds formed in electrode fouling processes. In a real‐life example, over‐oxidation products from phenolic cross‐coupling reaction on BDD anodes could be traced.

## Conflict of interest

The authors declare no conflict of interest.

## Supporting information

As a service to our authors and readers, this journal provides supporting information supplied by the authors. Such materials are peer reviewed and may be re‐organized for online delivery, but are not copy‐edited or typeset. Technical support issues arising from supporting information (other than missing files) should be addressed to the authors.

Supplementary
