# Supplementary material for: Mass‐Spectrometric Imaging of Electrode Surfaces—a View on Electrochemical Side Reactions
Source: Angew Chem Int Ed Engl. 2020 Sep 2;59(46):20428–33. doi: 10.1002/anie.202010134 (PMC7693111; doi:10.1002/anie.202010134)
Supplement: Supplementary file 1 — Supplementary [file ANIE-59-20428-s001.pdf]

Angewandte Chemie
Eine Zeitschrift der Gesellschaft Deutscher Chemiker

Supporting Information

Mass-Spectrometric Imaging of Electrode Surfaces—a View on Electrochemical Side Reactions

*Jens Fangmeyer[+], Arne Behrens[+], Barbara Gleede, Siegfried R. Waldvogel, and Uwe Karst**

anie_202010134_sm_miscellaneous_information.pdf

SI-1: Mass spectrometric parameters for the analysis of the three organic compounds via MALDI-ToF-MS

All mass spectra and imaging data were recorded using a timsTOF fleX (Bruker Daltonik, Bremen, Germany), equipped with a 355 nm Nd:YAG SmartBeam 3D laser.

A: aniline

MALDI(+); imaging laser mode with 100 µm spot size; laser energy, 70%; laser frequency, 1000; shots, 100; MALDI plate offset, 50 V; funnel 1 RF, 280.0 Vpp; funnel 2 RF, 500.0 Vpp; isCID energy, 0.0 eV; multipole RF, 350.0 Vpp; deflection delta, 220.0 V; quadrupole ion energy, -5.0 eV; low mass, mass-to-charge-ratio (*m*/*z*) 50.0; collision energy, 5.0 eV; collision RF, 300.0 Vpp; transfer time, 70.0 µs; pre pulse storage, 10.0 µs; *m*/*z* 100-1500.

B: 4-ethylphenol

MALDI(-); imaging laser mode with 100 µm spot size; laser energy, 70%; laser frequency, 1000; shots, 100; MALDI plate offset, -50 V; funnel 1 RF, 350.0 Vpp; funnel 2 RF, 550.0 Vpp; isCID energy, 0.0 eV; multipole RF, 320.0 Vpp; deflection delta, 220.0 V; quadrupole ion energy, -5.0 eV; low mass, mass-to-charge-ratio (*m*/*z*) 30.0; collision energy, -5.0 eV; collision RF, 300.0 Vpp; transfer time, 70.0 µs; pre pulse storage, 8.0 µs; *m*/*z* 100-1500.

C: *o*-phenylenediamine

MALDI(+); imaging laser mode with 100 µm spot size; laser energy, 70%; laser frequency, 1000; shots, 100; MALDI plate offset, 50 V; funnel 1 RF, 280.0 Vpp; funnel 2 RF, 500.0 Vpp; isCID energy, 0.0 eV; multipole RF, 350.0 Vpp; deflection delta, 220.0 V; quadrupole ion energy, 5.0 eV; low mass, mass-to-charge-ratio (*m*/*z*) 50.0; collision energy, -5.0 eV; collision RF, 570.0 Vpp; transfer time, 70.0 µs; pre pulse storage, 10.0 µs; *m*/*z* 100-1500.

D: phenol-phenol cross coupling

MALDI(-); imaging laser mode with 100 µm spot size; laser energy, 20%; laser frequency, 1000; shots, 50; MALDI plate offset, -50 V; funnel 1 RF, 350.0 Vpp; funnel 2 RF, 550.0 Vpp; isCID energy, 0.0 eV; multipole RF, 320.0 Vpp; deflection delta, 220.0 V; quadrupole ion energy, -5.0 eV; low mass, mass-to-charge-ratio (*m*/*z*) 30.0; collision energy, -5.0 eV; collision RF, 300.0 Vpp; transfer time, 70.0 µs; pre pulse storage, 8.0 µs; *m*/*z* 100-1500.

SI-2: Mass spectrometric parameters for the analysis of the three organic compounds via ESI-ToF-MS

All mass spectra were recorded using a timsTOF fleX (Bruker Daltonik, Bremen, Germany), using the ESI source of the instrument.

A: aniline

ESI(+); nebulizer (N_2), 0.3 bar; dry gas (N_2), 3.0 L/min; dry heater, 200 °C; capillary, 3.5 kV; endplate offset, -500 V; funnel 1 RF, 280.0 Vpp; funnel 2 RF, 500.0 Vpp; isCID energy, 0.0 eV; multipole RF, 350.0 Vpp; deflection delta, 220.0 V; quadrupole ion energy, -5.0 eV; low mass, mass-to-charge-ratio (*m*/*z*) 50.0; collision energy, 5.0 eV; collision RF, 300.0 Vpp; transfer time, 70.0 µs; pre pulse storage, 10.0 µs; *m*/*z* 100-1500.

B: 4-ethylphenol

ESI(-); nebulizer (N_2), 0.3 bar; dry gas (N_2), 3.0 L/min; dry heater, 200 °C; capillary, 2.5 kV; endplate offset, -500 V; funnel 1 RF, 350.0 Vpp; funnel 2 RF, 550.0 Vpp; isCID energy, 0.0 eV; multipole RF, 320.0 Vpp; deflection delta, 220.0 V; quadrupole ion energy, -5.0 eV; low mass, mass-to-charge-ratio (*m*/*z*) 30.0; collision energy, -5.0 eV; collision RF, 300.0 Vpp; transfer time, 70.0 µs; pre pulse storage, 8.0 µs; *m*/*z* 100-1500.

C: *o*-phenylenediamine

ESI(+); nebulizer (N_2), 0.3 bar; dry gas (N_2), 3.0 L/min; dry heater, 200 °C; capillary, 3.5 kV; endplate offset, -500 V; funnel 1 RF, 280.0 Vpp; funnel 2 RF, 500.0 Vpp; isCID energy, 0.0 eV; multipole RF, 350.0 Vpp; deflection delta, 220.0 V; quadrupole ion energy, 5.0 eV; low mass, mass-to-charge-ratio (*m*/*z*) 50.0; collision energy, -5.0 eV; collision RF, 570.0 Vpp; transfer time, 70.0 µs; pre pulse storage, 10.0 µs; *m*/*z* 100-1500.

SI-3: Sample preparation, electrochemical oxidation, matrix application, data analysis

Chemicals were purchased commercially and utilized without further purification steps. For all compounds we prepared solutions of 10 mM concentration in a mixture of 10 mM ammonium formate as electrolyte in water and acetonitrile with a ratio of 80/20 (v/v). For aniline and *o*-phenylenediamine, the electrolyte solution was optimized to be at pH 7, for 4-ethylphenol pH 9 was adjusted. pH value adjustment was achieved with aqueous ammonia (25%) solution.

DIACHEM® BDD electrodes were purchased from Condias GmbH (Itzehoe, Germany). Electrodes were of tantalum substrate covered with a 7 µm BDD multilayer from both sides with an overall thickness of 1 mm. The material was cut in 12 x 30 mm electrodes by a water jet cutting plant.

The electrodes were used as working electrode in a thin-layer flow-through cell (µ-PrepCell 2.0, Antec Scientific, Zoeterwoude, The Netherlands). The cell was operated with a constant flowrate of 10 µL/min. For all three compounds, the optimal potential was determined by introducing 10 µM aliquots of the respective stock solutions from SI-1 in the MS-instrument via its electrospray ionization (ESI) source. An external in-house developed potentiostat was used to apply a potential ramp in a window from 0 to 2.5 V with 10 mV/s. From the obtained MS data set, three-dimensional mass voltammograms were plotted to determine the best oxidation potential for each compound. The respective mass voltammograms are shown in the following figures:

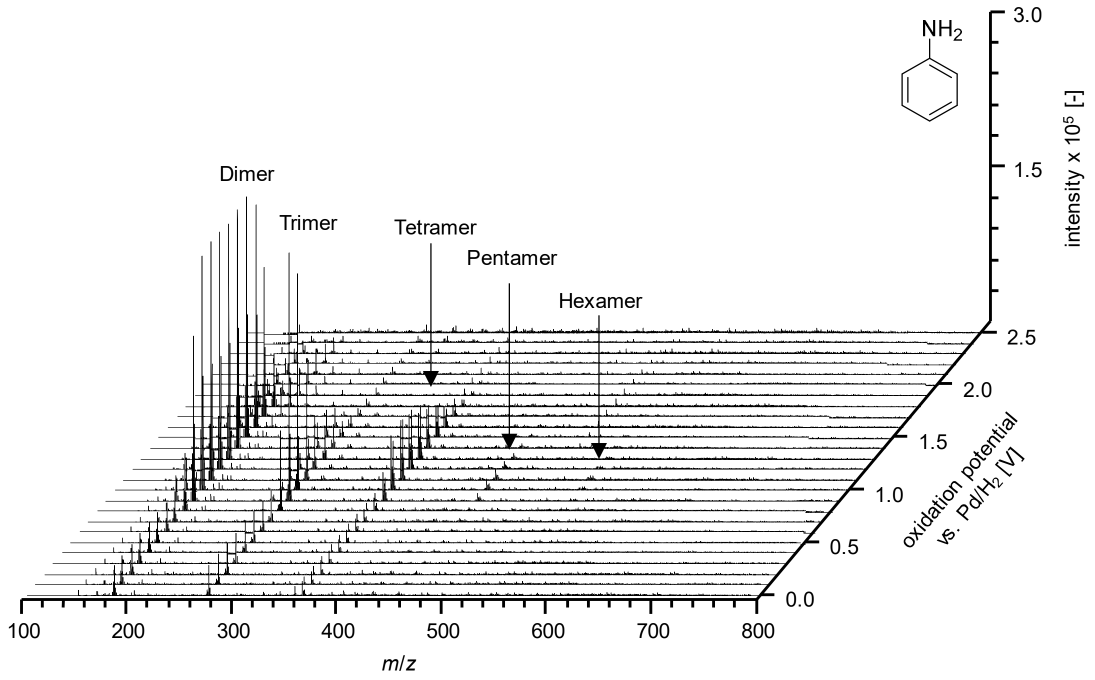

Figure SI-3a: Mass voltammogram for the oxidative potential optimization of aniline. Potential was determined to be most suitable for further experiments at 1 V.

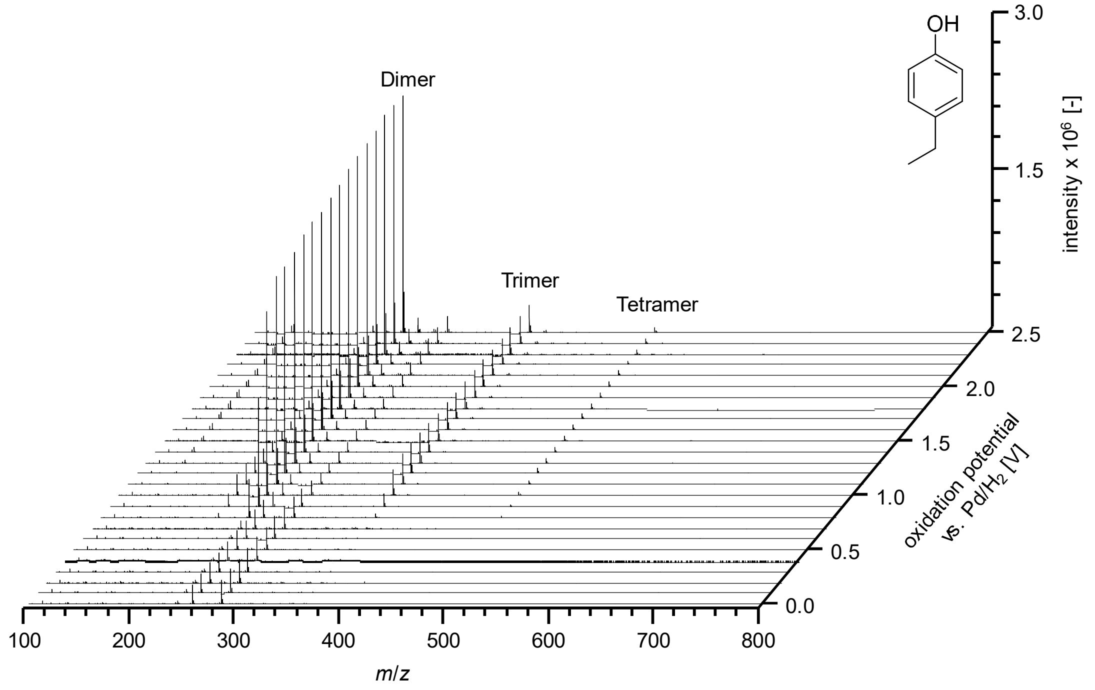

Figure SI-3b: Mass voltammogram for the oxidative potential optimization of 4-ethylphenol. Potential was determined to be most suitable for further experiments at 2 V.

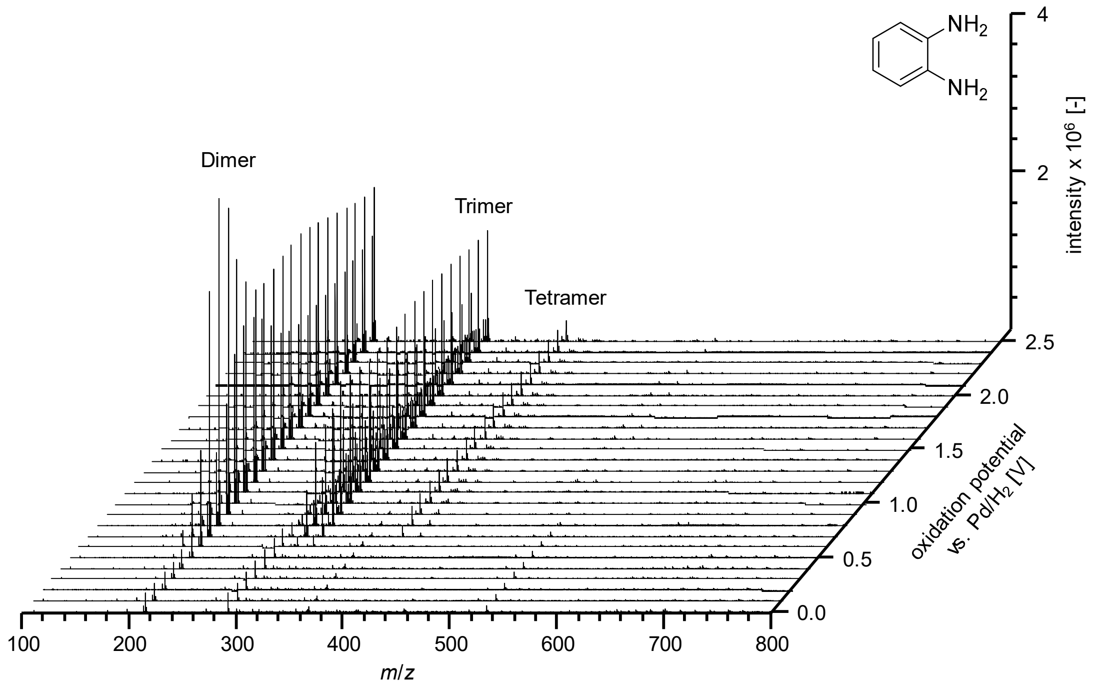

Figure SI-3c: Mass voltammogram for the oxidative potential optimization of *o*-phenylenediamine. Potential was determined to be most suitable for further experiments at 2 V.

Three electrodes were used with the optimized potentials (aniline, 1 V; 4-ethylphenol, 2 V; *o*-phenylenediamine, 2 V) for one hour each in the flow-through arrangement. Afterwards, the cell was disassembled and the remaining compound solution on the electrode surface was washed away by dipping the electrode in bidistilled water. 2,5-Dihydroxybenzoic acid (DHB) was used as matrix for the analysis of the aniline electrode. Therefore, we used a solution of 10 mg/mL DHB in a mixture of water and methanol (50%/50%, v/v). For matrix application, a TM-Sprayer (HTX Technologies LLC, Chapel Hill, NC, USA) was utilized. The other two compounds were analyzed with an LDI approach without matrix.

Mass spectral images were processed and plotted with the software SCiLS lab (Bruker Daltonik GmbH, Bremen, Germany).

SI-4: ESI-MS spectra of electrochemical oxidized compounds

The mass spectra represent a 10 µM direct infused aliquot of the 10 mM oxidized stock solution of the respective compounds analyzed via ESI-MS. For aniline (Figure SI-4a), only dimeric and trimeric structures can be observed. For 4-ethylphenol (Figure SI-4b) and *o*-phenylenediamine (Figure SI-4c), oligomers with up to four monomeric units were detected.

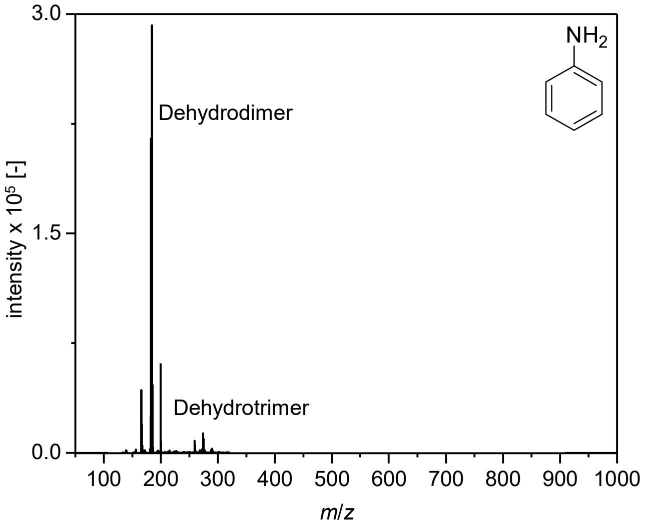

Figure SI-4a: ESI mass spectrum of a 10 µM direct infused aniline solution from an oxidized stock solution with 10 mM.

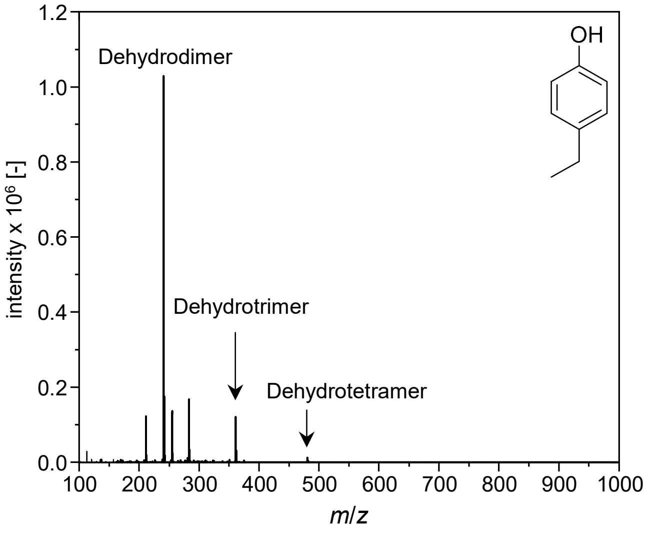

Figure SI-4b: ESI mass spectrum of a 10 µM direct infused 4-ethylphenol solution from an oxidized stock solution with 10 mM.

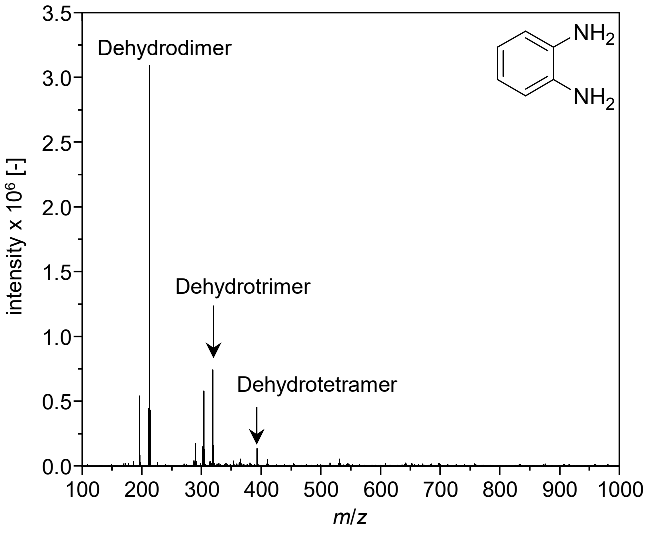

Figure SI-4c: ESI mass spectrum of a 10 µM direct infused *o*-phenylenediamine solution from an oxidized stock solution with 10 mM.

SI-5: Dehydrogenative cross-coupling of phenols

General information

All reagents were used in analytical grades. Solvents were purified by standard methods.[1] As supporting electrolyte *N*-methyl-*N*,*N*,*N*-tributylammonium methylsulfate (kindly provided by BASF SE, Ludwigshafen, Germany) were employed.

Column chromatography was performed on silica gel 60 M (0.040–0.063 mm, Macherey-Nagel GmbH & Co, Düren, Germany) with a maximum pressure of 1.6 bar. As eluents mixtures of cyclohexane and ethyl acetate were used. Silica gel 60 sheets on aluminum (F254, Merck, Darmstadt, Germany) were employed for thin layer chromatography.

Gas chromatography was performed on a Shimadzu GC-2010 (Shimadzu, Japan) using a ZB-5 column (Phenomenex, USA; length: 30 m, inner diameter: 0.25 mm, film: 0.25 mm, carrier gas: hydrogen). GC-MS measurements were carried out on a Shimadzu GC-2010 (Shimadzu, Japan) using a ZB-5 column (Phenomenex, USA; length: 30 m, inner diameter: 0.25 mm, film: 0.25 mm, carrier gas: hydrogen). The method was coupled with mass spectrometry on a Shimadzu GCMS-QP2010.

Melting points were determined with a Melting Point Apparatus M-565 (Büchi, Essen, Germany) and were uncorrected. Heating rate: 2 °C/min.

^{1}H NMR spectra were recorded at 25 °C by using a Bruker Avance III HD 400 (400 MHz; 5 mm BBFO-SmartProbe with z gradient and ATM, SampleXPress 60 sample changer, Analytische Messtechnik, Karlsruhe, Germany). Chemical shifts (δ) are reported in parts per million (ppm) relative to TMS as internal standard or traces of $CHCl_3$ in the corresponding deuterated solvent. Mass spectra are obtained by using a MAT 95 (Finnigan, Germany) apparatus employing FD.

BDD electrodes

For electrochemical conversion, boron-doped diamond electrodes (BDD) with a 15 µm diamond coating and siliconized carbon fiber carbon composite (CFC) as support material were applied. BDD electrodes were obtained from CONDIAS GmbH (Itzehoe, Germany) using hot filament technology (HF-CVD). The exact pre-treatment and preparation are reported.[2] BDD electrodes were used for several electrolysis, which were published before.[3]

Synthesis of 2‘,4-dihydroxy-3,5-dimethoxy-5‘-(1,1-dimethylethyl)biphenyl

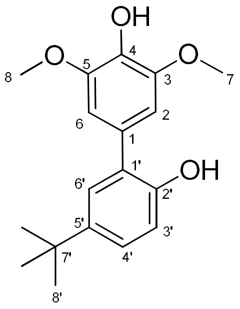

A solution of 2,6-dimethoxyphenol (0.12 g, 0,76 mmol, 1.0 eq.), 4-(1,1-dimethylethyl)phenol (0,34 g, 2,27 mmol, 3.0 eq.) and *N*-methyl-*N*,*N*,*N*-tributylammonium methylsulfate (0.15 g, 0.45 mmol) were dissolved in 5 mL 1,1,1,3,3,3-hexafluoro-2-propanol (HFIP) or HFIP with 18 vol.% methanol and transferred into an undivided beaker-type cell equipped with BDD-electrodes. A constant current electrolysis with a current density of 7.2 mA/cm² was performed at 50 °C. After application of 146 C the electrolysis was stopped and the solvent mixture was recovered *in vacuo* (50 °C, 200–70 mbar). The crude product was purified by column chromatography (cyclohexane/ethyl acetate (4 : 1)). The desired product was obtained as colorless solid. (mp: 159 °C (cyclohexane/ethyl acetate (4 : 1)); R_f: 0.23 (cyclohexane/ethyl acetate (4 : 1)); MS (FD): *m/z* = 302.3 (calculated for M: $C_{18}H_{22}O_4$ = 302.3)

^{1}**H NMR** (400 MHz, $CDCl_3$): δ [ppm]= 7.29 (dd, $^{3,4}J$ = 8.5, 2.5 Hz, 1H, *H*-4‘), 7.24 (d, ^{4}J = 2.5 Hz , 1H, *H*-6‘), 6.94 (d, ^{3}J = 8.5 Hz, 1H, *H*-3‘), 6.68 (s, 2H, *H*-2, *H*-6), 3,92 (s, 6H, *H*-7, *H*-8), 1.34 (s, 9H, *H*-8‘).

Analytical data agree with those previously reported.[4,5]

SI-6: Additional LDI-MS images of over-oxidized A2B-type dehydrotrimers

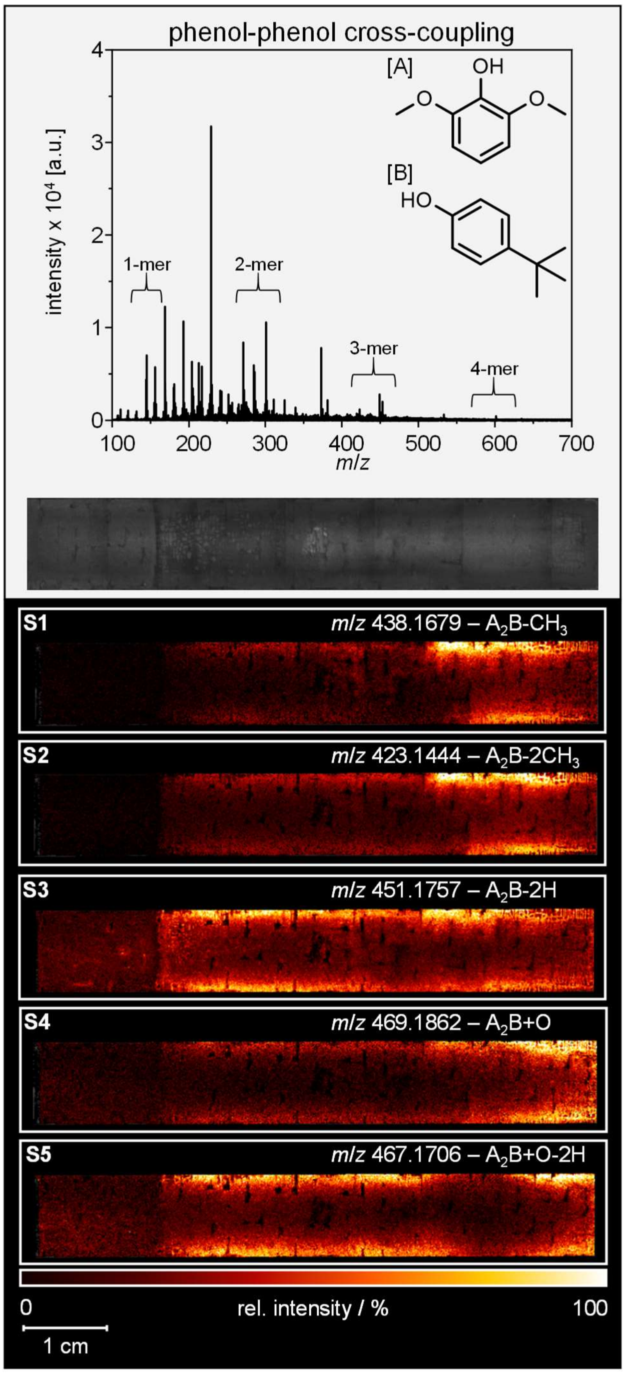

Figure SI-6: Mean mass spectrum (negative ion mode) for the BDD electrode of the phenol-phenol cross-coupling reaction. Optical image of the electrode after electrochemical treatment. Below: Selected LDI-MS images of the spatial distribution of over-oxidized A2B-type dehydrotrimers of oxidative treated 2,6-dimethoxyphenol (A) and (1,1-dimethylethyl)phenol (B; R1-5). The flow direction for the electrochemical conversion is from the left to the right.

References:

[1] W. L. F. Armarego, C. L. L. Chai; Purification of laboratory chemicals, Elsevier, Amsterdam, **2013**.

[2] a) M. Fryda, T. Mattheé, S. Mulcahy; CONDIAS GMBH (Ed.). Method of applying a diamond to a graphite substrate. EP2077344 (B1).; b) M. Fryda, T. Mattheé; CONDIAS GMBH (Ed.). Diamond electrodes and methods for its production; c) H. Puetter, A. Weiper-Idelmann, C. Merk; CONDIAS GMBH (Ed.). Diamond coated electrodes. EP1036861 (A1). 2000.

[3] B. Gleede, T. Yamamoto, K. Nakahara, A. Botz, T. Graßl, R. Neuber, T. Matthée, Y. Einaga, W. Schuhmann, S. R. Waldvogel, *ChemElectroChem.* **2019**, 6, 2771–2776.

[4] A. Libman, H. Shalit, Y. Vainer, S. Narute, S. Kozuch, D. Pappo, *J. Am. Chem. Soc.* **2015**, *137*, 11453–11460.

[5] B. Riehl, K. Dyballa, R. Franke, S. Waldvogel, *Synthesis* **2016**, *49*, 252–259.
